# Concurrent Outbreak of Norovirus Genotype I and Enterotoxigenic *Escherichia coli* on a U.S. Navy Ship following a Visit to Lima, Peru

**DOI:** 10.1371/journal.pone.0020822

**Published:** 2011-06-21

**Authors:** Victor E. Gonzaga, Mariana Ramos, Ryan C. Maves, Randal Freeman, Joel M. Montgomery

**Affiliations:** 1 United States Naval Medical Research Unit Six (NAMRU-6), Lima, Peru; 2 United States Army, El Paso, Texas, United States of America; University of Texas Medical Branch, United States of America

## Abstract

An outbreak of norovirus (NoV) genotype I and Enterotoxigenic *Escherichia coli* (ETEC) occurred among US Navy Ship personnel following a visit to Lima, Peru, in June 2008. Visiting a specific area in Lima was significantly associated with illness. While ETEC and NoV are commonly recognized as causative agents of outbreaks, co-circulation of both pathogens has been rarely observed in shipboard outbreaks.

## Introduction

The discovery of novel gastrointestinal viruses has prompted the global health community to reevaluate their epidemiological perspective on the common causes of gastroenteritis. Norovirus (NoV) is responsible for approximately 50% of all food-borne gastroenteritis outbreaks in the United States [1]. Outbreaks related to NoV have usually been reported in closed settings [2], [3]. Currently, there are five recognized NoV genogroups, of which three (I,II and IV) are known to cause illness in humans [4]. NoV infection often presents as acute-onset vomiting, watery non-bloody diarrhea with abdominal cramps and nausea. The incubation period ranges from 24–48 hours and is typically transmitted through direct person-to-person contact or through the oral fecal route [4]. For this reason NoV is frequently associated with large outbreaks within military populations and on military and civilian ships, reaching attack rates as high as 60% [2]. Several studies have attempted to estimate the prevalence of NoV and other viral infections among diarrheal cases in Latin America [5], [6] but the knowledge is still limited and much more information is needed to fully understand the burden of illness. Co-circulation of NoV with enterotoxigenic *Escherichia coli* (ETEC) as cause of outbreaks has been poorly described on the literature [7]. On June 30th, 2008 an outbreak of a gastrointestinal illness occurred aboard a US Navy ship in the port of Lima. Several days prior to the outbreak, numerous personnel visited tourist destinations and restaurants throughout Lima. An investigation was conducted to identify the etiologic agent, to evaluate factors associated with the outbreak and to provide recommendations to the ship's commander on how to control the current and prevent future outbreaks.

## Methods

The study protocol was approved by the U.S. Naval Medical Research Unit Six (NAMRU-6) Institutional Review Board in compliance with all applicable Federal regulations governing the protection of human subjects. NAMRU-6's IRB determined that this project did not meet the definition of human subject research and waived the need for consent; therefore we could proceed without further review by IRB. This study was based on a response to an outbreak of Norovirus in a ship and involved testing of human specimens previously obtained by the ship's medical staff.

An unmatched case-control study was conducted among crew members on June 30^th^, 2008. A case was defined as an individual within the crew with a history of vomiting and/or diarrhea from June 21^st^ until July 10^th^, 2008. One control per case was randomly selected from the unaffected crew population. A self administered questionnaire was completed by both cases and controls. Socio-demographic, clinical and epidemiological variables were assessed, including history of contact with ill individuals, hand washing behaviors and exposure to a specific location known as “Pizza Alley”, which was frequently reported by cases during preliminary descriptive studies. Stool and emesis samples were collected from eleven symptomatic patients and tested for evidence of bacterial, parasitic and NoV infection. Light microscopy was used for parasite identification, while conventional culture procedures were used for bacterial identification, followed by multiplex PCR to differentiate pathogenic strains of *E. coli* [8]. Real time RT-PCR was employed to detect NoV using primers and probes for the polymerase gene of both genotypes I and II (GI and GII), as previously described [9]. Initial RNA extracts were purified prior to direct sequencing on an ABI Prism 3130 sequencer (Applied Biosystems) using cycle sequencing dye terminator chemistry (Perkin-Elmer, Foster City, CA). Sequences were compared against published sequences using NCBI's Basic Local Alignment Search Tool (BLAST; available at http://www.ncbi.nlm.nih.gov/blast/Blast.cgi). Odds ratios (OR) and 95% confidence intervals (CI) were calculated using bivariate and multivariate logistic regression analysis in STATA 11.0.

## Results

**Figure 1:**
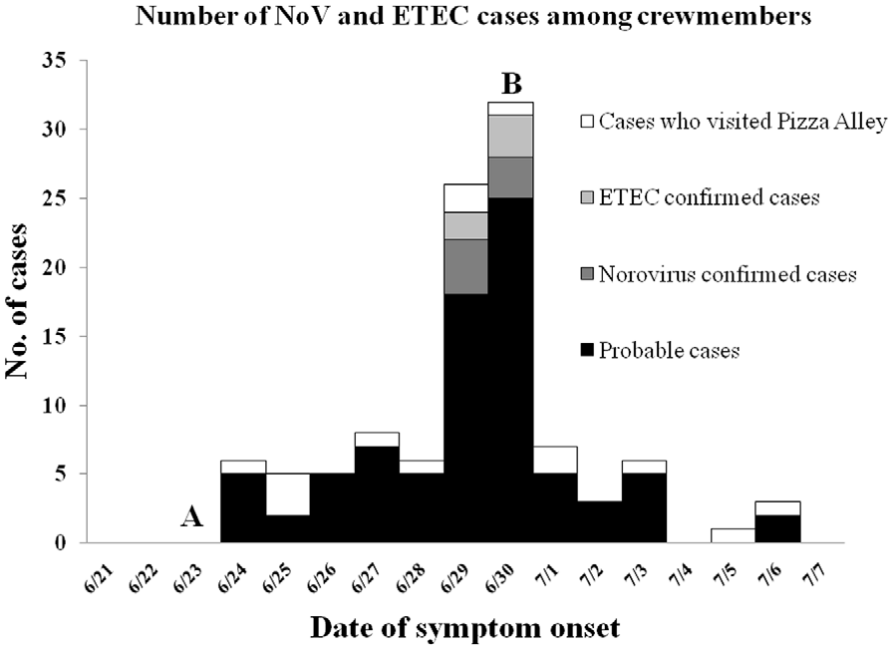
Number of cases of acute gastroenteritis among 230 members of the crew showing the evolution of the outbreak of NoV GI and ETEC in a US Navy ship. A. Crew members visited several locations during their visit to Lima, including “Pizza Alley” a location of many restaurants, bars and nightclubs. B. Control measures included: thorough cleaning of all ship surfaces with a 5% bleach solution, reinforcement of the importance of proper hand washing, provision of hand sanitizers (alcohol hand gel) in each room, recommendation to only consumed cooked foods (including vegetables) and requirement for any crewmember to report to the ships infirmary in the event they develop symptoms of gastroenteritis.

One hundred and thirty individuals of the 230 total crewmembers were interviewed on July 1^st^, 2008 (response rate: 57%; 65 cases: 65 controls). NoV GI was identified in 1 emesis sample and 9/11 (82%) stool samples. Enterotoxigenic *E. coli* (ETEC) was also detected by PCR in 8/11 (73%) stool samples, although cultures for *E. coli* were negative. Of the 11 samples tested, 6 were positive to both ETEC and NoV. All symptomatic individuals had been treated with ciprofloxacin prior to sample collection. Viral sequence was 96% homologous to NoV GI. The clinical attack rate was 28.3%; mean age of individuals was 28.7 years; 85.3% were male. We observed no difference in mean age and gender between groups (p = 0.6 and p = 0.07, respectively). The peak of the outbreak occurred 7 days after the index case began with symptoms (Figure 1). Most frequent symptoms included diarrhea (93.9%), nausea (66.2%), abdominal cramps (65.3%) and vomiting (40%). Mean duration of symptoms was 2.7±1.4 days. Several factors associated with illness were identified in the bivariate analysis (Table 1). We separately evaluated the consumption of particular food such as vegetables, fruits, raw fish or seafood; however, no association was observed. Only two variables remained significant in the multivariate analyses, after adjusting for contact with a case and self-reporting of hand washing after being in contact with a case. Factors associated with an increased risk of infection included, visiting a specific location (“Pizza Alley”) in Lima (OR = 8.04, IC95% 1.22 to 52.73; p = 0.03). Consumption of only bottled water during the visit to Peru was shown to be highly protective against infection (OR = 0.01, IC95% 0.001 to 0.17; p = 0.001).

**Table 1:** Exposures and activities associated with acute gastroenteritis.

Exposure or activity	Case patients (n = 65)	Control patients (n = 65)	OR (95%CI)	P
Age (median; range)	28 (23–32)	30 (24–33)	-	0.49
Gender: male	59 (90.8%)	52 (80%)	0.39 (0.14 to 1.12)	0.08
**Bivariate analysis**				
Visiting a specific area in Lima¶	14/28 (50%)	5/48 (10.42%)	8.60 (2.62 to 28.15)	<0.001
Having contact with a case§	36 (56.25%)	14 (21.88%)	4.59 (2.12 to 9.93)	<0.001
Consumption of vegetables during their stay in Lima€	57 (87.69%)	50 (76.92%)	2.62 (1.01 to 6.80)	0.05
Participating in a humanitarian mission to Ventanilla*	5 (7.69%)	7 (10.77%)	0.69 (0.20 to 2.29)	0.54
Hand washing after using the ship's gym	52 (86.67%)	57 (90.48%)	0.68 (0.22 to 2.10)	0.5
Hand washing after having had contact with a case§	31 (48.44%)	43 (68.25%)	0.43 (0.21 to 0.89)	0.02
Hand washing after using the restroom	3 (4.69%)	7 (10.77%)	0.40 (0.10 to 1.65)	0.20
Consumption of bottled water during stay in Lima	5 (7.69%)	40 (62.50%)	0.05 (0.01 to 0.14)	<0.001
**Multivariate analysis**				
Visiting a specific area in Lima¶	-	-	8.04 (1.22 to 52.73)	0.03
Consumption of bottled water during stay in Lima	-	-	0.01 (0.001 to 0.17)	0.001

**NOTE:** OR, odds ratio; CI, 95% confidence intervals.

¶ Numerous crewmembers (including the index cases(s)) visited several locations during their visit to Lima, including “Pizza Alley,” a location of many restaurants, bars and nightclubs.

§ i.e. Sharing a berthing facility, being in close proximity during daily activities, etc.

€ Both cooked and uncooked vegetables.

*Ventanilla is a low income area of Lima near the Port of Callao, Peru. A group of crewmembers conducted housing construction and renovation during one day of their deployment to Lima, as part of a humanitarian assistance mission.

## Discussion

This study describes a gastrointestinal outbreak which occurred on a US Navy ship in the port of Callao, Peru. Although NoV GI and ETEC were both identified as etiological agents in the early phase of the outbreak, it is likely that NoV was the source of the outbreak in the secondary wave based upon its mechanism of transmission. Retrospective investigations have identified NoV GI as the cause of outbreaks and single infections among populations of several South and Central American countries [3], [5], including a recent extensive outbreak which occurred in Antofagasta, Chile [10]. This is the first description of an outbreak related to infection with NoV GI in Peru and, to our knowledge, the first outbreak of NoV reported on a US naval ship in South America. With the exception of one study conducted in Denmark, outbreaks linked to dual infections of NoV and ETEC have been limited and poorly described [7]. Although we were unable to collect control samples, which may have helped to better define the role of ETEC in this outbreak, our data suggests NoV was likely the primary cause of illness in the later cases due to its short incubation period and daily presentation of new cases several days after leaving port, suggesting person-to-person transmission. Unlike NoV, there is some evidence suggesting that ETEC is not readily transmitted via fomites [11], this may be due to a shorter half-life of the pathogen in the environment. However, ETEC and NoV have been shown to be co-associated in individuals with diarrhea and among cases in outbreaks [10], [12]. The dual role of these pathogens in outbreaks and illness should be carefully evaluated in future investigations.

NoV infection is a frequent cause of diarrhea and vomiting among travelers to developing countries [13] and has been widely recognized as an important cause of outbreaks on US military and civilian ships [2], [14]. Although outbreaks on US Navy ships are not typically associated with visits to high-risk ports for traveler's diarrhea [14], our findings demonstrate that NoV has the potential to be introduced into a ship following personnel visits to a foreign port during deployed missions. Precautions among the ship's personnel (i.e., health education on food safety and proper food and hand hygiene) should be reinforced prior to entering port cities. Precautions should also include avoiding the consumption of beverages prepared with unknown source water, including ice, during these visits. The protective role of consuming bottled water observed in our study may have been related to individuals who were less likely to engage in risky behaviors such as ingestion of uncooked foods or poor hand hygiene, both of which are known to be linked to and propagate food-borne outbreaks. Personnel should be encouraged to drink only bottled beverages, consume thoroughly washed or cooked foods and routinely wash their hands prior to eating.

In this outbreak, the primary infection (i.e., index case(s)) likely occurred during their visit to a specific area in Lima, with subsequent person-to-person and fomite transmission (i.e., hand rails, bunks, etc.). Although the initial disinfection of the ship probably removed the majority of the surface contamination, repeated contamination of these surfaces likely occurred, as 28% of the crewmembers were symptomatic and moving throughout the ship. Persistence of NoV is among the most significant contributing factors for shipboard outbreaks, as surfaces can remain contaminated for lengthy periods of time facilitating transmission of the infection to naïve individuals. Thus, various prevention measures should be considered to minimize dissemination of this virus during shipboard outbreaks. Such measures may include, a shipboard outbreak response program which could allow implementation of rapid and effective control measures during gastrointestinal outbreaks (i.e., proper disinfection guidelines), informational materials providing symptomatic individuals with guidance on personal hygiene and recommendations on temporary self-isolation/movement restriction, informational materials for asymptomatic individuals on how to prevent illness during an outbreak (i.e., hand hygiene, etc.) and installation of alcohol hand gel dispensers in key locations. Finally, surveillance and identification of gastrointestinal viruses, including NoV GI, during gastrointestinal outbreaks and among individuals suffering from gastrointestinal illness should be considered, to better define the epidemiology and burden of illness caused by these viruses and their interaction with other pathogens (e.g., ETEC) in developing countries, particularly in South America.
